# Compartmental diffusion and microstructural properties of human brain gray and white matter studied with double diffusion encoding magnetic resonance spectroscopy of metabolites and water

**DOI:** 10.1016/j.neuroimage.2021.117981

**Published:** 2021-07-01

**Authors:** Henrik Lundell, Chloé Najac, Marjolein Bulk, Hermien E. Kan, Andrew G. Webb, Itamar Ronen

**Affiliations:** a Danish Research Centre for Magnetic Resonance, Copenhagen University Hospital Hvidovre, Centre for Functional and Diagnostic Imaging and Research, Kettegaards Allé 30, 2650 Hvidovre, Denmark; b C.J. Gorter Center for High Field MRI, Department of Radiology, Leiden University Medical Center, Albinusdreef 2, 2333 ZA Leiden, The Netherlands

**Keywords:** Double diffusion encoding, Magnetic resonance spectroscopy, Human brain tissue, Microscopic anisotropy, Compartment specificity

## Abstract

Double diffusion encoding (DDE) of the water signal offers a unique ability to separate the effect of microscopic anisotropic diffusion in structural units of tissue from the overall macroscopic orientational distribution of cells. However, the specificity in detected microscopic anisotropy is limited as the signal is averaged over different cell types and across tissue compartments. Performing side-by-side water and metabolite DDE spectroscopic (DDES) experiments provides complementary measures from which intracellular and extracellular microscopic fractional anisotropies (μFA) and diffusivities can be estimated. Metabolites are largely confined to the intracellular space and therefore provide a benchmark for intracellular μFA and diffusivities of specific cell types. By contrast, water DDES measurements allow examination of the separate contributions to water μFA and diffusivity from the intra- and extracellular spaces, by using a wide range of *b* values to gradually eliminate the extracellular contribution. Here, we aimed to estimate tissue and compartment specific human brain microstructure by combining water and metabolites DDES experiments. We performed our DDES measurements in two brain regions that contain widely different amounts of white matter (WM) and gray matter (GM): parietal white matter (PWM) and occipital gray matter (OGM) in a total of 20 healthy volunteers at 7 Tesla. Metabolite DDES measurements were performed at *b* = 7199 s/mm^2^, while water DDES measurements were performed with a range of *b* values from 918 to 7199 s/mm^2^. The experimental framework we employed here resulted in a set of insights pertaining to the morphology of the intracellular and extracellular spaces in both gray and white matter. Results of the metabolite DDES experiments in both PWM and OGM suggest a highly anisotropic intracellular space within neurons and glia, with the possible exception of gray matter glia. The water μFA obtained from the DDES results at high *b* values in both regions converged with that of the metabolite DDES, suggesting that the signal from the extracellular space is indeed effectively suppressed at the highest *b* value. The μFA measured in the OGM significantly decreased at lower *b* values, suggesting a considerably lower anisotropy of the extracellular space in GM compared to WM. In PWM, the water μFA remained high even at the lowest *b* value, indicating a high degree of organization in the interstitial space in WM. Tortuosity values in the cytoplasm for water and tNAA, obtained with correlation analysis of microscopic parallel diffusivity with respect to GM/WM tissue fraction in the volume of interest, are remarkably similar for both molecules, while exhibiting a clear difference between gray and white matter, suggesting a more crowded cytoplasm and more complex cytomorphology of neuronal cell bodies and dendrites in GM than those found in long-range axons in WM.

## Highlights

• Compartment and cell-specific microscopic anisotropy are measured with DDES.
• The intracellular space is highly anisotropic in white (WM) and gray matter (GM).
• The extracellular space in GM is isotropic, while that of WM is highly anisotropic.
• Water and metabolites intracellular mean diffusivities are lower in GM than in WM.
• Intracellular tortuosity derived from water and tNAA is higher in GM than WM.

## Graphical abstract

**Figure:**
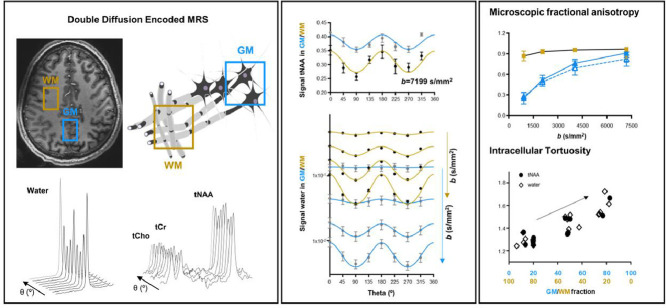

## Introduction

Diffusion-weighted MRI (DW-MRI) sensitizes the signal to molecular displacement on length scales comparable to cell morphological features, making DW-MRI a sensitive tool for tissue characterization on a microscopic scale Johansen-Berg and Behrens (2013). Inferring specific cytomorphological properties from DW-MRI is not straightforward, as diffusion measurements reflect the average properties across a large volume compared to cellular dimensions. This average reflects properties that range from the microscopic shape and size of restricting geometries of different cell types to the heterogeneous organization over the entire voxel. Some microstructural information may be retrieved from modeling conventional diffusion data from Stejskal-Tanner experiments (Stejskal and Tanner, 1965), thereby accounting for the heterogeneity across the imaging voxel. This, however, heavily relies on model assumptions that are difficult to validate with the ambiguous and sparse histological data available (Jones et al., 2013, Novikov et al., 2018).

**Fig. 1:**
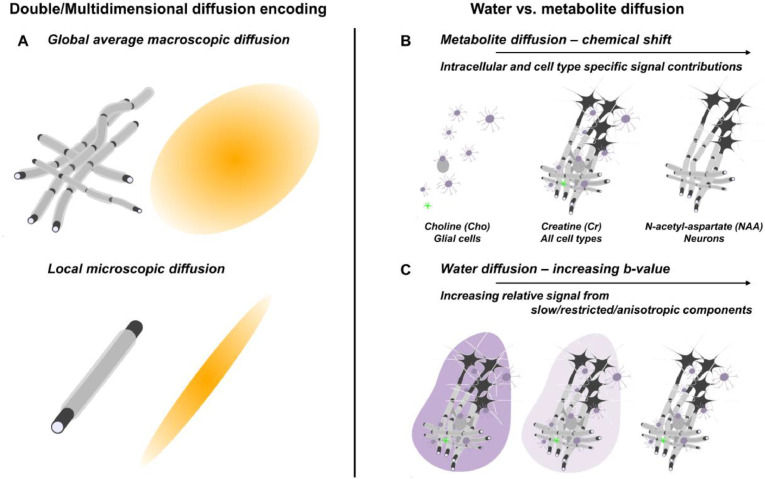
Approaches used in this study to provide compartment-specific readouts of diffusivity: (A) Double diffusion encoding (DDE) enables the decoupling of global effects of fibers across the acquisition volume from local microscopic diffusion. (B) Studying metabolite diffusion with DDES provides access to cell-preferential or specific intracellular microscopic organization, including cell bodies and fibrous processes of the respective cell types. (C) Measuring water diffusion with DDES over a large range of *b* values allows separating the properties of CSF, the intra- and extracellular spaces. The progressively fading purple background indicates the gradual elimination of extracellular and CSF signals with increasing *b* value.

A different approach is to increase specificity to the underlying cytomorphological features at the data acquisition stage. Several methods have been suggested to separate anisotropic diffusion on the *microscopic* scale, reflecting the presence of thin fibers such as axons and astrocytic processes, from *macroscopic* anisotropy, which reflects co-alignment of fibers across the acquisition volume. These methods include double diffusion encoding (DDE) experiments (Shemesh et al., 2016, Henriques et al., 2020), related tensor valued encoding methods in multiple directions with tailored gradient waveforms Topgaard (2017), and analysis of the non-monoexponential attenuation of disordered samples or the so-called “powder average” of diffusion measurements in multiple directions with respect to the *b* value in conventional diffusion experiments (Callaghan et al., 1979, Kroenke et al., 2004, Palombo et al., 2017, Lundell et al., 2020, Kaden et al., 2016, (Veraart et al., 2019)). Microscopic axial (D_//_) and transverse (D_┴_) diffusivities as well as the related microscopic anisotropy (µFA) can be calculated from these measurements, and these reflect the diffusion properties within cytomorphological units on the length scale of the diffusion process, regardless of their mutual orientation on larger length scales (Fig. 1A) (Jespersen et al., 2013, Lasič et al., 2014, Jespersen, 2014). In contrast to inferring µFA from conventional data, DDE provides a unique fingerprint of anisotropic diffusion that reduce the risk of misinterpretation of other effects, such as non-monoexponential attenuation from multiple compartments (Henriques et al., 2019). Compartmental and cell-type selectivity, and in some cases specificity, can be obtained with diffusion-weighted magnetic resonance spectroscopy (DW-MRS) (Palombo et al., 2017, Ronen and Valette, 2015), where the diffusing microstructural probes are intracellular metabolites. DW-MRS is thus not only specific to the intracellular space but also allows studying cytomorphology of different cell populations such as neurons and glia across species (Fig. 1B) (Choi et al., 2007). The compartmental specificity of DW-MRS extends beyond obtaining standard diffusional metrics such as metabolite apparent diffusion coefficients (ADC). The combination of DDE and DW-MRS (DDES) (Shemesh et al., 2017, Vincent et al., 2020, Lundell et al., 2018, Najac et al., 2019) can yield cell-type-specific microscopic diffusion metrics of the intracellular space, and contribute to a more detailed characterization of cytomorphology in neural tissue.

The morphological properties of the intracellular and extracellular spaces are not independent of one another, especially in white matter (WM), where the dominant structural unit is long-range axons in WM tracts. The relatively compact packing of axons in WM suggests the possibility of microscopic anisotropy also in the extracellular space (Sen and Basser, 2005, Fieremans et al., 2016). However, work interpreting conventional DW-MRI with biophysical models suggests that microscopic anisotropy is considerably lower in the extracellular space than in the intracellular space (Zhang et al., 2012, Assaf and Basser, 2005, Jespersen et al., 2007, Novikov et al., 2018). Tailored measurements with either isotropic diffusion weighting or with modulation of the extracellular signal fraction with contrast agents suggest similar mean diffusivities of the extra and intracellular spaces (Kunz et al., 2018, Dhital et al., 2017, Lampinen et al., 2016). This makes the isolation of either of these compartments difficult to achieve at low *b* values. The signal at higher *b* values, however, will prevail only for spins in highly anisotropic components angulated with respect to the encoding gradient (Callaghan et al., 1979). This has inspired the notion of using large diffusion weightings as a filter to target measurements of intra-axonal water ((Veraart et al., 2019), Budde et al., 2017, Dhital et al., 2019, Tax et al., 2020) (Fig. 1C). For a quantitative assessment of the elimination of extracellular water in our experiments, see supplementary material S1.

The microstructural characterization of both the intracellular and the extracellular spaces in gray matter (GM) has been less investigated. The microstructural heterogeneity of human GM, in addition to the lack of evidence of macroscopic anisotropy except for that found in neonates (McKinstry et al., 2002) and some cortical regions (McNab et al., 2013), has discouraged researchers from further exploring the microstructural properties of GM in detail with DW-MRI. Pioneering work with DDE demonstrated the presence of microscopic anisotropy in GM of excised pig spinal cord and brain samples (Komlosh et al., 2008, Komlosh et al., 2007, Shemesh and Cohen, 2011). Only recently have attempts been made to characterize microscopic anisotropy in human GM *in vivo*, either with tensor valued encoding techniques (Lampinen et al., 2016) or with DDE (Lawrenz and Finsterbusch, 2019).

In this study, we present a DDES approach to characterize the microscopic anisotropy and diffusivities of cell type specific intracellular spaces and the extracellular spaces in GM and WM of the human brain. The complementarity of the DDES water measurements at low *b* values, which includes contributions from both the intra- and extracellular spaces, with the intracellular-specific DDES measurements of metabolites and water at high *b* values results in a set of unique quantitative insights on the morphology of the intracellular space in gray and white matter. In addition, qualitative assessments of the microstructural characteristics of the extracellular space are also derived.

## Materials and methods

### Human subjects

A total of 20 healthy volunteers (age 33±12 years, 11 females) participated in the study, with 2 volunteers participating twice for acquisitions in two different regions (see description below). The study adhered to the guidelines of the Leiden University Medical Center Institutional Review Board (The Netherlands). Informed consent was obtained from all subjects prior to the session.

### MRI scanner/hardware

All experiments were performed on a 7T Philips Achieva whole-body MRI scanner (Philips Healthcare, Best, The Netherlands) equipped with a volume transmit/32-channel receive head coil (Nova Medical, Wilmington MA, USA) and gradient coils with a maximum gradient strength of 40 mT/m and a slew rate of 200 T/m/s. A high permittivity dielectric pad (suspension of barium titanate in D_2_O) was used to maximize the transmit magnetic field (B_1_^+^) homogeneity and efficiency in the parietal and occipital regions as previously described (Snaar et al., 2011, Ingo et al., 2018).

### MRI/DW-MRS data acquisition

The acquisition time for the entire protocol averaged approximately 55 minutes and comprised the following scans.

#### Anatomical images

**Fig. 2:**
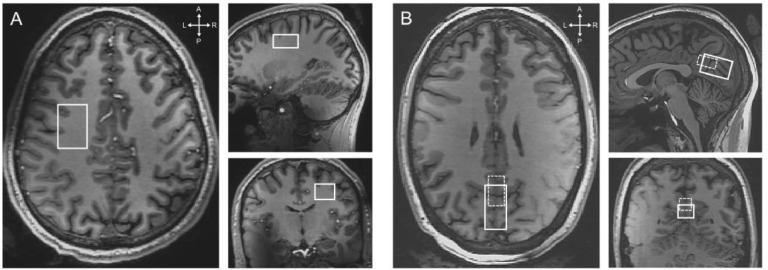
Representative placement in the of the DDES VOI in (A) the PWM region and (B) the OGM region. The smaller OGM VOI (dashed lines) was added to increase the GM fraction within the VOI.

A short survey scan and a sensitivity encoding (SENSE) reference scan followed by a 3D T_1_-weighted gradient-echo acquisition were conducted to allow for the planning of the volumes-of-interest (VOIs) for the DDES experiments (Fig. 2). Imaging parameters for the 3D-T_1_ weighted scan were: field-of-view (a-p, f-h, r-l): 246 × 246 × 174 mm^3^, in-plane resolution: 1 × 1 × 1 mm^3^, repetition time (TR)/echo time (TE): 4.9/2.2 ms, flip angle: 7°, total scan duration: 1:55.

#### Water and metabolite DDES

**Fig. 3:**
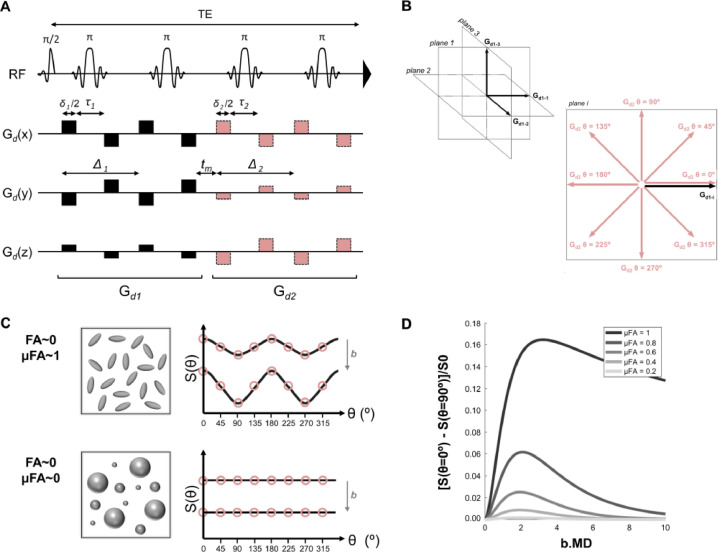
(A) Schematic drawing of the DDE-sLASER sequence. For simplicity, only diffusion sensitizing gradients are shown. (B) Illustration of diffusion weighting (DW) gradient directions used in our study. The direction of the first DW group (G_d1_, black vector) is fixed along one direction (larger panel to the right), while the direction of the second diffusion group (G_d2_, shaded vectors) revolves in 8 angular steps in a plane that includes G_d1_. This measurement is repeated for 3 orthogonal directions of G_d1_ with G_d2_ revolving in 3 separate orthogonal planes. (C) Schematic illustration of the modulation of the signal as a function of θ (angle between first and second encoding) expected for ensembles of rotationally disperse anisotropic (top) or isotropic (bottom) diffusion tensors for two different *b* values. (D) The contrast between parallel (θ = 0°) and perpendicular (θ = 90°) encodings for a rotationally uniform distribution of monodisperse diffusion tensors with different µFA as a function of the unitless attenuation factor *b*.MD (MD=(D_//_ +2D_┴_)/3, Mean Diffusivity).

*Pulse sequence*: DDES data were acquired using a DDE-sLASER sequence (Boer et al., 2011). The sequence diagram is shown in Fig. 3A. The following acquisition parameters were used: TE: 185 ms, spectral width: 3000 Hz, number of time-domain points: 1024. Each of the two diffusion weighting modules within our DDES sequence consisted of a double spin-echo with a bipolar diffusion weighting scheme. Using the conventions established for DDE sequences (Shemesh et al., 2016) adapted to a bipolar DW scheme: for both DW modules, single gradient lobe duration *δ_1_*/2 = *δ_2_*/2 = 15.5 ms; bipolar gap *τ_1_ =* *τ_2_* = 10 ms; gradient separation time Δ_1_ = Δ_2_ = 45 ms; mixing time t_m_ = 5.3 ms (see Fig. 3A for definitions of timing parameters).

*Volumes of interest*: We examined two different brain regions: a WM region within the left parietal lobe (PWM) and a GM cortical region within the occipital lobe (OGM). 9 subjects were scanned with a 9 cm^3^ VOI in the PWM region (3 cm (a-p), 2 cm (f-h), 1.5 cm (r-l)); 9 subjects were scanned with a 9 cm^3^ VOI in the OGM region (3 cm (a-p), 1.5 cm (f-h), 2 cm (r-l)). To increase the specificity to GM in the OGM, 4 subjects were scanned with a smaller OGM VOI (sOGM) of 2.5 cm^3^ (2.5 cm (a-p), 1 cm(f-h), 1 cm (r-l)). Two subjects participated twice: one was scanned with PWM and OGM VOIs and another was scanned with OGM and sOGM VOIs. Due to the limited SNR, only water acquisition was possible in the smaller VOI. The positions of the VOIs are illustrated in Fig. 2. Due to time constraints, metabolites and water DDES scans at all *b* values were also not acquired in all subjects. Table 1 summarizes the sample size for each acquisition performed in PWM and OGM VOIs.

**Table 1:** Sample size (n) for water and metabolite DDES experiments in PWM and OGM VOIs with the range of *b* values used.

	Water DDES			Metabolites DDES	
	PWM	OGM	sOGM	PWM	OGM
*b* = 918 s/mm^2^	6	8	4		
*b* = 2066 s/mm^2^	6	6	4	6	7
*b* = 4050 s/mm^2^	7	6	4		
*b* = 7199 s/mm^2^	8	7	4		

*Water suppression, B_0_shimming, and scan synchronization:* VOI-localized B_0_ shimming up to second order was performed. To minimize signal fluctuations due to cardiac pulsation, cardiac triggering was achieved using a peripheral pulse unit (trigger delay: 250 ms, TR: 5 cardiac cycles). For metabolite acquisitions, water suppression was achieved using two frequency-selective excitation pulses, each followed by a dephasing gradient.

*DDE acquisition schemes*: The angular DDE encoding was performed in three orthogonal planes spanned by the three vectors [1 1 -0.5], [-0.5 1 1], and [1 -0.5 1]. These directions provide the maximum combined gradient amplitude for three orthogonal directions. For each acquisition, the first encoding direction was fixed and the second encoding was performed in 8 equally spaced directions tracing a full circle, starting with the first encoding direction (see Fig. 3B). θ refers to the angle between the effective gradient directions of the first and second encoding, adhering to the definition given in Shemesh et al. (2016). Two diffusion gradient amplitudes were used for metabolite acquisitions, resulting in 2 *b* values for the entire diffusion encoding scheme: 0 and 7199 s/mm^2^. Five diffusion gradient amplitudes, resulting in 5 *b* values (0, 918, 2066, 4050, and 7199 s/mm^2^) were used for water acquisitions. To compensate for cross-terms between diffusion and imaging/background gradients (Neeman et al., 1991) in the post-processing stage, DW data were acquired alternately with opposite diffusion gradient polarities. Overall, each DW condition was repeated 6 times for metabolites and 2 times for water acquisitions. For metabolite DDES, the total number of acquisitions was N_acq_ = (3 (directions first encoding) x 8 (directions second encoding) x 2 (gradient polarities) + 1 (*b* = 0 condition)) x 6 (number of averages) = 294 acquisitions. With TR of 5 cardiac cycles, the total scan time was about 294 × 5 = 25 min. For water DDES, the number of averages was 2, resulting in N_acq_ = 98 and an 8 min scan.

#### Data processing and analysis

 

#### Image processing

T_1_-weighted images were segmented into tissue maps for GM, WM and cerebrospinal fluid (CSF) using FSL (Brain extraction Tool Smith (2002) and FAST (Zhang et al., 2001) algorithm in the FMRIB Software Library). Each voxel contains a value in the range 0-1 that represents the proportion of each tissue. An in-house Matlab routine (MathWorks, Inc., Ma, USA) was then used to quantify the tissue volumes within each spectroscopic VOI. One subject was excluded from the tissue analysis due to the poor quality of the segmentation.

#### DDES Data pre-processing

**Fig. 4:**
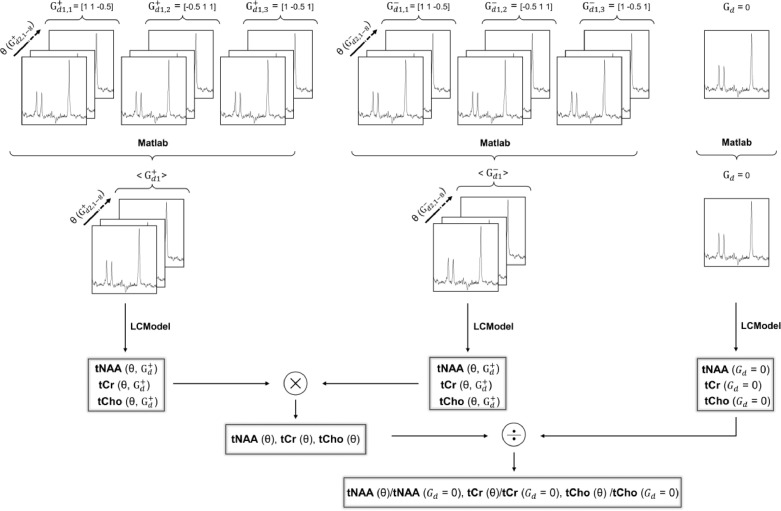
Illustration of metabolite data pre-processing. Metabolite data sets consisted of individual spectra acquired with (*b* = 7199 s/mm^2^) and without (*b* = 0 s/mm^2^) diffusion-weighting (DW). DW spectra were alternatingly acquired with opposite gradient polarity ($G_{d}^{+}$ and $G_{d}^{-}$) and with 24 angular conditions (3 (G_d1_) x 8 (G_d2_)). DW spectra were first powder-averaged across the three G_d1_ values. DW and non-DW spectra were then quantified with LCModel (Provencher, 2001) resulting in signal amplitudes of tNAA, tCr, and tCho. The geometric means of estimates with opposite polarity were finally calculated and normalized to their respective non-DW signals.

Individual spectra were corrected for eddy currents, phase, and frequency drifts using in-house Matlab routines as previously described (Kan et al., 2012). Averaged metabolite data sets consisted of 49 individual spectra: one acquired with *b* = 0 s/mm^2^ and two sets of 24 (3 (G_d1_) x 8 (G_d2_)) DW spectra at *b* = 7199 s/mm^2^, each set acquired with two gradient polarities. DW spectra were subsequently averaged across the three G_d1_ directions to provide an emulated powder average, resulting in two sets of 8 DW spectra. These spectra and the one acquired at *b* = 0 s/mm^2^ were subsequently quantified with LCModel (Provencher, 2001), resulting in signal amplitudes of total N-acetyl-aspartate (tNAA = N-acetyl aspartate (NAA) + N-acetylaspartylglutamate (NAAG)), total creatine (tCr = creatine (Cr) + phosphocreatine (PCr)) and total choline (tCho = choline (Cho) + phosphocholine (PCho) + glycerophosphocholine (GPC)) for each value of *b*, θ and gradient polarity. The LCModel basis set included a total of 16 metabolites and a control node spacing of the spline function for fitting the baseline (dkntmn) of 0.5. Finally, the geometric mean of the LCModel estimates was calculated for each pair of spectra acquired with the same (*b*, θ) acquired with opposite sign of the gradient polarity, thereby reducing the effect of cross-terms with background and sequence gradients (Neeman et al., 1991). The geometric mean is defined as $\bar{S_{geom.}}=\sqrt{S^{+}\cdot S^{-}},$ where S^+^ and S^−^ are the LCModel estimates of the signal of a given metabolite obtained with the positive and negative polarities of the DW gradients, respectively. An evaluation of this effect on phantom data is shown in supplementary figure S2. The resulting diffusion-weighted metabolite signals were finally normalized to their respective signal at *b* = 0 s/mm^2^. The post-processing scheme is depicted in Fig. 4.

The water signal was preprocessed similarly, and water spectra acquired with the same diffusion-weighted condition (*b*, θ, gradient polarity) were averaged. The amplitude of the water signal for each averaged spectrum was obtained by integrating the area under the water peak in Matlab. The remaining post-processing procedure for the water data at each of the 4 *b* values followed the one described above for the metabolites, resulting in a single water signal value for *b* = 0 s/mm^2^ and 4 sets of 8 water signal values, for each value of *b* and θ, respectively. All DW water signals were normalized to the signal at *b* = 0 s/mm^2^. A short representation of the raw data is available in the supplementary tables S2 and S3. The raw data and associated analysis code is available from Itamar Ronen (i.ronen@lumc.nl) upon request and data handling agreement.

### Analyses and interpretation of DDES data

Under the assumption of mono-disperse, axially symmetric diffusion tensors D with axial and radial diffusivities D_//_ and D_┴,_ the *b* value and θ-dependent DDE signal from an ensemble of *N* different orientations described by the rotation matrices $R_{j}$ can be calculated as:

$$\begin{array}{ccc} S\left(b,\theta \right) & = & \frac{S_{0}}{N}\sum_{j=1}^{N}\text{exp}\left(-\frac{b}{2}e_{1}R_{j}{\mathrm{DR}}_{j}^{T}e_{1}^{T}\right)\\ & & \text{exp}\left(-\frac{b}{2}\left(cos^{2}\theta e_{1}R_{j}{\mathrm{DR}}_{j}^{T}e_{1}^{T}+sin^{2}\theta e_{2}R_{j}{\mathrm{DR}}_{j}^{T}e_{2}^{T}\right)\right) \end{array}$$

The two orthogonal unit vectors $e_{1}$ and $e_{2}$ span the encoding plane. The signal from a powder average was emulated with *N* = 256 uniformly distributed rotations with respect to the axial direction of an axially symmetric diffusion tensor. The two exponentials in the summation reflect the signal attenuation from the first and second diffusion encoding gradient acting on each rotation $R_{j}$ of the diffusion tensor D. While the signal of a powder average at low *b* values reflects the initial slope or the mean diffusivity of the ensemble, the different angular modulation of anisotropic components with different orientations gives a multiexponential behavior that becomes more apparent at higher *b* values (shown schematically in Fig. 3C and simulated for monodisperse diffusion tensors in Fig. 3D).

For metabolites, the single compartment diffusivity was estimated from the *b* = 0 s/mm^2^ normalized signal ($S\left(b,\theta \right)/S_{0}$) with the axial (D_//_) and radial (D_┴_) microscopic diffusivities as fitting parameters. $S_{0}$ was not fitted, as only one datapoint at *b* = 0 s/mm^2^ is available after averaging and spectral quantification. A similar assumption for the water signal is not expected to be valid, as multiple signal components from CSF and different intra- and extracellular environments violate the monodisperse assumption. However, residual restricted and anisotropic components would be expected to dominate the signal at larger *b* values. Here, the water signal was fitted using data from two subsequent *b* values with $S_{0}$ as an additional fitting parameter to extrapolate the signal at *b* = 0 s/mm^2^ with reduced contributions from more isotropic compartments such as CSF. For mixed components with well-separated diffusivities, the fitted $S_{0}$ at high *b* values thus reflects the residual volume of slow and anisotropic signal components. Some hypothetical considerations for signal contributions from different environments are shown in the supplementary figure S1. All fitting procedures were performed using non-linear sum of squares error minimization. We constrained the fit to prolate geometries (D_//_ > D_┴_) and initially found identical results with random initializations. Final fit to the data was performed with D_//_ = 1 µm^2^/ms, D_┴_ = 0 and $S_{0}$ = 1 (fitting parameter for water and fixed for metabolites) as initial values. S_0_ was a fitting parameter for water and was kept fixed for metabolites. Reported mean values and standard deviations of parameter estimates are from individual estimates of each participant, but fits to the average signal across all participants were performed to show the modulation of the signal as a function of θ. The microscopic fractional anisotropy (µFA) was calculated analogous to the fractional anisotropic (FA) from DTI analyses:

$$\mu \text{FA}=\sqrt{\frac{{\left(D_{//}-D_{⊥}\right)}^{2}}{D_{//}^{2}+2D_{⊥}^{2}}}$$

### Statistical analysis

Results are expressed as mean ± standard deviation. Statistical significance was tested using GraphPad Prism 7 using an unpaired Student's *t* test with unequal variance (GraphPad Software, USA). Statistical tests for the comparisons of S_0_, D_//,_ D_┴,_ and μFA between PWM and OGM VOIs were corrected for multiple comparisons using a false discovery rate approach using the Benjamini-Hochberg method. Adjusted p values are reported for a false discovery rate of 5%. A threshold of *p* < 0.05 was considered significant, the following symbols where used to indicate the significance: *p < 0.05, **p < 0.01, ***p < 0.001, ****p ≤ 0.0001. Correlation between intracellular tortuosity and D_//_ with tissue fraction were obtained with linear regression using GraphPad Prism 7. Here we corrected for multiple comparisons using the Bonferroni method with an adjusted significance level for the 4 independent tests with the significance threshold set to *p* < 0.05/4 = 0.0125.

## Results

### Volume Fraction of GM, WM, and CSF

Table 2 shows the average percentages of WM, GM, and CSF within our two VOIs. The average WM fraction in the PWM VOI is above 80% while the average GM fraction is about 15%. In comparison, the average WM fraction in the OGM VOI is lower than 40% and the average GM fraction is around 50%. To increase the specificity to GM, a subset of data was acquired in a smaller OGM VOI (sOGM), which contained on average around 70% GM and 20% WM. GM and WM content were significantly different in OGM VOI compared to PWM VOI (*p* < 0.0001). Finally, the CSF fraction was fairly low in the PWM VOI but was significantly higher in OGM VOI (~14%, *p* < 0.006) as it included the interhemispheric fissure. While the CSF signal does not contribute to the diffusion results at high *b*, it influences the results at the lower *b*, particularly in the OGM VOI.

**Table 2:** Volume fraction (%, mean±s.d.) of WM, GM, and CSF in the parietal white matter (PWM), occipital gray matter (OGM) and small occipital gray matter (sOGM) VOIs. Statistical significance between PWM and OGM VOIs (represented with *) and between GM and WM content (represented with ‡) were evaluated using an unpaired Student's t-test with *, ^‡^*p*<0.05, **, ^‡‡^*p*<0.005, ***, ^‡‡‡^*p*<0.001 and ****, ^‡‡‡‡^*p*<0.0001.

	PWM VOI	OGM VOI	sOGM VOI	PWM *vs*. OGM
Gray Matter (GM)	15.1±5.1 ^‡‡‡‡^	50.1±7.9 ^‡^	67.7±4.5 ^‡‡‡‡^	****
White Matter (WM)	82.8±5.7	36.2±15.1	19.8±3.9	****
Cerebrospinal Fluid (CSF)	2.1±1.2	13.7±9.1	12.5±6.4	**

### Intracellular microscopic D_//_, D_┴_ and μFA from metabolite DDES data

**Fig. 5:**
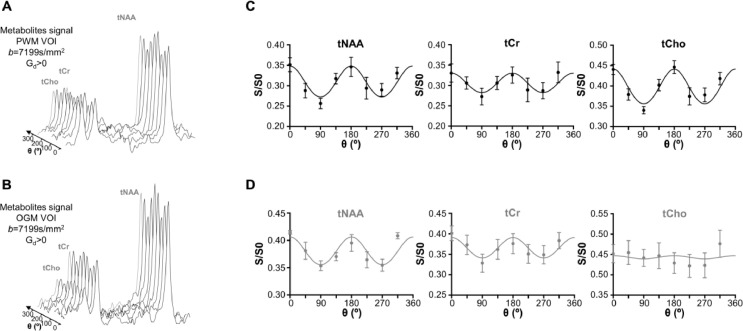
Illustration of individual DDES metabolite spectra for different θ in (A) PWM and (B) OGM VOIs. Following signal quantification, the θ-modulation for tNAA, tCr, and tCho in both (C) PWM (black) and (D) OGM (gray) VOIs were fitted using an ensemble of uniformly rotated axisymmetric diffusion tensors. The data (circle) and fits (solid line) for the mean over all participants are illustrated for both VOIs, and the error bars indicate the standard error of the mean.

**Fig. 6:**
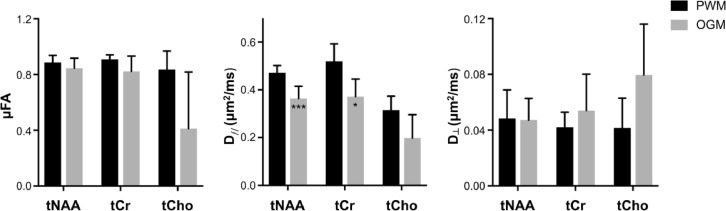
Fitted model parameters for the metabolites data (mean±s.d.). Statistical significance between PWM and (large) OGM (represented with *) and between tCho and tNAA (represented ‡) in both VOIs was evaluated using an unpaired Student's t-test with unequal variance and a false discovery rate correction for multiple comparison. *, ^‡^*p* < 0.05, **, ^‡‡^*p* < 0.005 and ***, ^‡‡‡^*p* < 0.001.

Representative metabolite DDES spectra from the PWM and OGM VOIs are shown as a function of θ in Figs. 5A and 5B. The resonances of tNAA, tCr and tCho could be identified and reliably quantified using LCModel in both regions. Cramér–Rao lower bounds (PWM/OGM) for the spectral fit (as percent of the standard deviation) were 4±1/4±1, 10±2/8±2 and 8±1/12±3 for tNAA, tCr, and tCho respectively. Values refer to measurements at *b*=7199 s/mm^2^ and were averaged across subjects and values of θ. Panels C and D in Fig. 5 show the fitting results of the θ-modulated signal (averaged over subjects) for the PWM and the OGM VOIs. Fig. 6 reports the averaged values for metabolite D_//,_ D_┴,_ and μFA and the results of the statistical analysis. For tNAA and tCr, D_//_ was significantly lower in OGM compared to PWM (*p* ≤ 0.023). D_┴_ and μFA were not significantly different between the two VOIs for all three metabolites. Finally, μFA > 0.8 for all three intracellular metabolites in both VOIs, except for tCho in OGM where a lower µFA was found accompanied by both an increase in D_┴,_ and a reduction in D_//_ . Differences in all three tCho diffusion metrics between PWM and OGM did not reach statistical significance.

### D_//_, D_┴_ and μFA from water DDES data over a range of b values

#### Water DDES at the highest b value

**Fig. 7:**
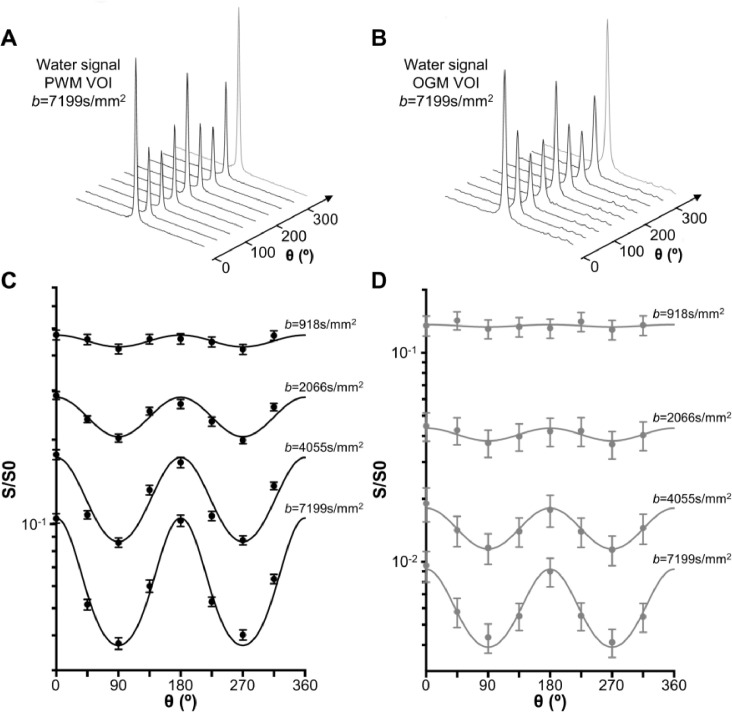
Individual water spectra (geometric mean of positive and negative gradient polarities) for different θ in (A) PWM and (B) OGM VOIs. Following signal quantification, the θ-modulation for both (C) PWM and (D) OGM VOIs was fitted using an ensemble of uniformly rotated axisymmetric diffusion tensors. The data (circles) and fits (solid line) for the mean over all participants are illustrated for both VOIs, error bars indicate the standard error.

The water signal was quantified over a range of *b* values in the PWM and OGM VOIs (Fig. 7). Table 3 reports the averaged values and statistical analyses for S_0_, D_//,_ D_┴,_ and μFA. S_0_ was evaluated from each consecutive pair of *b* values and normalized to the S_0_ at the lowest *b* (918 s/mm^2^) as explained in the methods section, and is referred to as “fitted S_0_” from here on. At the highest *b*, the fitted S_0_ was significantly lower in OGM VOI compared to PWM VOI. In both VOIs, μFA(water) > 0.8 at the highest *b*. μFA(water) and D_//_(water) were significantly higher in the PWM VOI than in the OGM VOI (*p* < 0.0002), while D_┴_(water) was significantly lower in the PWM VOI than in the OGM VOI (*p* < 0.0005). Finally, water diffusion metrics values obtained from the sOGM VOI are reported in the supplementary materials (table S1). Due to the small sample size, no statistical tests were performed between these values and those from the other two VOIs.

**Table 3:** Fitted model parameters for the water data (mean ± s.d.). Statistical significance was evaluated using an unpaired Student's t-test with unequal variance and a false discovery rate correction for multiple comparison. * denotes significance level in difference between PWM and OGM in the same table cell. ‡ denotes significance level in differences between the diffusion measure/VOI at the *b* value in that column and the same diffusion measure/VOI at the highest *b* value (*b* = 7199 s/mm^2^). *, ^‡^*p*<0.05, **, ^‡‡^*p*<0.005, ***, ^‡‡‡^*p*<0.001,****, ^‡‡‡‡^*p*<0.0001.

		*b*=918 s/mm^2^			*b*=2066 s/mm^2^			*b*=4050 s/mm^2^			*b*=7199 s/mm^2^		
Fitted S_0_	PWM	1.00			0.86±0.14	^‡‡^	***	0.74±0.09		****	0.65±0.10		****
	OGM	1.00			0.38±0.12	^‡‡‡^		0.16±0.07	^‡^		0.08±0.05		
D_//_(μm^2^/ms)	PWM	2.36±0.19	^‡‡‡‡^	*	2.07±0.08	^‡‡^	*	1.94±0.08		****	1.83±0.11		****
	OGM	2.90±0.34	^‡‡‡‡^		1.91±0.13	^‡‡‡‡^		1.56±0.12			1.43±0.13		
D_┴_(μm^2^/ms)	PWM	0.28±0.15	^‡‡^	****	0.13±0.06	^‡‡^	****	0.08±0.03		****	0.06±0.02		***
	OGM	1.93±0.34	^‡‡‡‡^		0.77±0.10	^‡‡‡‡^		0.32±0.06	^‡‡‡‡^		0.12±0.03		
μFA	PWM	0.87±0.07	^‡‡^	****	0.93±0.03	^‡‡^	****	0.96±0.01		****	0.97±0.01		***
	OGM	0.25±0.08	^‡‡‡‡^		0.52±0.07	^‡‡‡‡^		0.76±0.06	^‡‡‡^		0.91±0.03		

#### Water DDES across b values

**Fig. 8:**
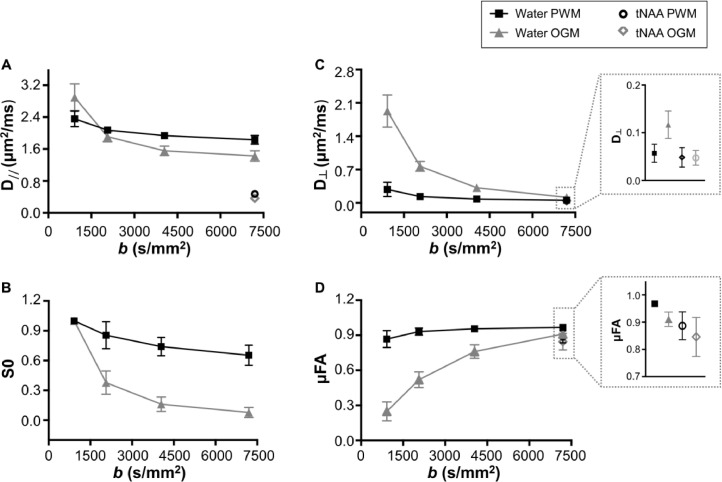
Water D_//_ (A), D_┴_ (B), S_0_ (C) and μFA (D) as a function of *b* value for the three brain regions: PWM VOI (black solid line) and OGM VOI (gray solid line). D_┴_ and μFA values for tNAA in PWM (back circles) and OGM (gray diamonds) VOIs at the highest *b* value are also displayed in the zoomed boxes.

We observed a significantly lower μFA(water) at all *b* < 7199 s/mm^2^ in OGM VOI (*p* < 0.0002) when compared to the μFA(water) at the highest *b* value (see Table 3 and Fig. 8). μFA(water) remained relatively unchanged in PWM across *b* values, with a significantly lower μFA(water) from *b* = 2066 s/mm^2^ (*p* < 0.010). A significantly higher D_//_(water) was observed at the two lowest *b* values (*p* < 0.001) when compared to the values at the highest *b* value in all VOIs. D_┴_(water) was also significantly higher at lower *b* values (from *b* ≤ 2060 s/mm^2^ in PWM and *b* ≤ 4050 s/mm^2^ in OGM VOIs, *p* < 0.005).

μFA(water) was significantly lower in OGM VOI compared to PWM VOI at all *b* (*p* < 0.0002). At the lowest *b*, μFA(water) was more than three times higher in PWM VOI compared to OGM VOI. D_//_(water) was significantly lower in OGM VOI compared to PWM VOI at *b* > 2066 s/mm^2^ and was higher at the lowest *b*. D_┴_(water) was significantly higher in OGM VOI compared to D_┴_(water) in the PWM VOI at all *b* (*p* < 0.0006).

### Getting down to the matter - D_//_ across molecules and the effect of partial volumes

**Fig. 9:**
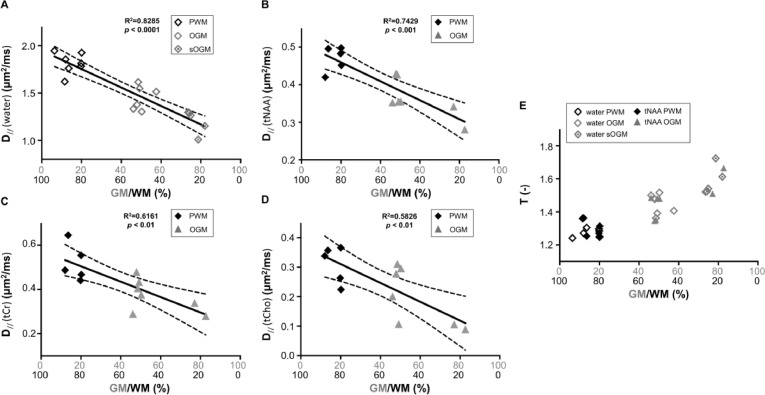
Correlation between GM/WM tissue fraction (%) and (A) water (B) tNAA (C) tCr, and (D) tCho parallel diffusivities (D_//_). Data for different VOIs are represented with different markers: PWM VOI as filled black diamonds, OGM and sOGM VOIs as filled and open gray triangles, respectively. The solid line shows the linear regression and the dashed lines represent the 95% confidence intervals. (E) Tortuosity values are estimated for tNAA (filled black circles) and water (open diamonds) as a function of GM/WM tissue fraction (%). The regression lines for the tortuosity are not displayed for clarity. R^2^ > 0.8 and *p* < 0.0002 for both tNAA and water. All tests are statistically significant with a corrected threshold of *p* < 0.0125.

Tissue fractions in both PWM and OGM VOIs widely varied across participants, thereby adding a significant source of variance to the diffusion metrics in each VOI. To address this source of variance we further investigated correlations between diffusion metrics and tissue fractions across all VOIs. Fig. 9 illustrates D_//_(tNAA) and D_//_(water) as a function of GM and WM content across subjects and brain regions. Tissue fractions were calculated with respect to the sum of GM and WM within the VOI, assuming that the contribution from CSF at the highest *b* is fully suppressed. D_//_(water) and D_//_(tNAA) increase with the fraction of WM in the VOI (*R^2^* = 0.83 and 0.74 respectively, *p* < 0.0003). Using reported values (Kroenke et al., 2004) for D_free_(water) (3 μm^2^/ms) and D_free_(tNAA) (0.78 μm^2^/ms) at 37°C, we estimated the intracellular tortuosity, T, for water and tNAA, where T = $\sqrt{D_{\text{free}}/D_{//}}$ (Fig. 9E), with extrapolated values at 100% WM and 100% GM presented in Table 4. For each tissue type (100% WM and 100% GM), similar tortuosity values for tNAA and water were obtained. Tortuosity values for OGM were significantly higher than those in PWM (*p* < 0.003 for both water and tNAA). D_//_ for tCr and tCho were also estimated, both indicating lower D_//_ for these metabolites in GM than in WM.

**Table 4:** Summary of all parameters extracted from the data acquired in PWM and OGM VOIs at highest *b* value (mean±s.d.).

			PWM	OGM	WM	GM
	GM (%)WM (%)		15.4±5.384.6±5.3	56.3±14.540.8±14.5	0100	1000

From modeling individual DDES data	D_//_ (μm^2^/ms)	*WatertNAA*	1.83±0.110.47±0.03	1.43±0.130.36±0.05		

	D_┴_ (μm^2^/ms)	*WatertNAA*	0.06±0.020.05±0.02	0.12±0.030.05±0.02		

μFA	*WatertNAA*	0.97±0.010.89±0.05	0.91±0.030.85±0.07		

T	*WatertNAA*	1.28±0.041.29±0.04	1.45±0.071.47±0.10		

From D_//_ and tissue fraction correlation	D_//_ (μm^2^/ms)	*WatertNAA*	1.80±0.120.47±0.04	1.40±0.260.41±0.08	1.95±0.060.51±0.02	0.97±0.170.25±0.07

T	*WatertNAA*	1.29±0.041.29±0.06	1.46±0.141.38±0.14	1.24±0.021.24±0.03	1.76±0.151.74±0.23

### Noise propagation in estimates of thin fibers

**Fig. 10:**
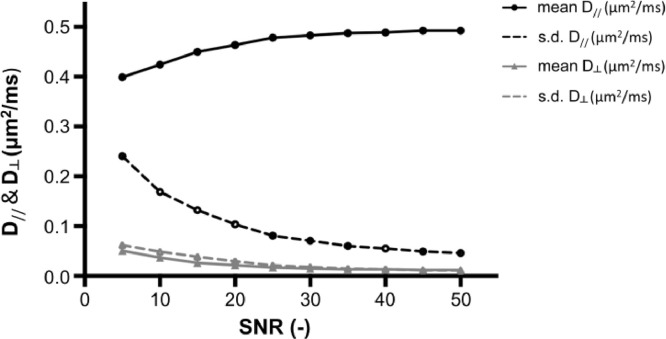
Noise propagation in the parameter estimates for settings comparable to the metabolite acquisition for a substrate with D_//_ = 0.5 µm^2^/ms and D_┴_ = 0 µm^2^/ms (“stick” situation) comparable to tNAA in white matter. Mean and standard deviations are estimated from 1000 realizations of the data with Gaussian noise at different SNR. Metabolite data presented in this paper has an SNR range ~30–45.

We evaluated the effect of SNR on the estimation of D_//_ and D_┴_ with a simulation that takes into account our experimental settings (Fig. 10). We assumed that D_┴_ = 0 (displacement RMS perpendicular to the fiber wall >> fiber diameter) and D_//_ = 0.5 µm^2^/ms which are representable values for tNAA (Lundell et al., 2020, Shemesh et al., 2017, Ronen et al., 2014). SNR is defined here as the tNAA signal relative to the standard deviation of the noise in the *b* = 0 condition. In our experiments, SNR for the tNAA peak estimated by LCModel was in the range of 30–45 for the data averaged across DW conditions. The simulation shown here indicates that D_┴_ of tNAA would be overestimated to about 0.02–0.03 µm^2^/ms, with a standard deviation on the same order.

## Discussion

In this work we demonstrate an approach for studying local microstructural features of brain tissue compartments by measuring and analyzing side-by-side water and metabolite DDES data. While metabolite DDES measurements provide an unequivocal empirical benchmark for intracellular diffusion metrics of neuronal and glial metabolites, water DDES measured with a range of *b* values enables the gradual elimination of the CSF and extracellular contributions, offering a reading of the intracellular diffusivity alone, as well as indirect information on the local geometry of the extracellular space. We used this approach to study the characteristics of intracellular diffusion processes of water and metabolites in GM and WM, highlighting common features as well as stark differences between the compartmental properties of these two tissue types. Table 4 summarizes the salient microstructural metrics obtained from the DDES measurements of metabolites and those of water at the highest *b*, highlighting our findings that pertain to the intracellular space in both GM and WM.

### Intracellular spaces are highly anisotropic but different in GM and WM

With the exception of μFA(tCho) in OGM, the μFA of all three metabolites was higher than 0.8 in both OGM and PWM. At the highest *b*, where it is assumed that faster-diffusing extracellular and CSF contributions are suppressed, μFA(water) was also very high (~0.9) and comparable with μFA(tNAA) and μFA(tCr) in both regions, as well as with μFA(tCho) in WM. This strongly suggests that in the time scale of our measurements, the intracellular space is highly anisotropic in both GM and WM.

This is evident in WM, where the high μFA for both intracellular water and the three metabolites suggests a predominant neurite morphology for all cell types. Our observation of a highly anisotropic intracellular space in WM supports the hypothesis of the prevalence of thin fibers in WM, which could consist of long-range axons or processes of fibrous astrocytes. The high μFA of the preferentially glial metabolite tCho suggests that the characteristic distance between branching points in WM glia exceeds the diffusion length in our DDES experiments, and within the diffusion/mixing time of our experiments. tCho experiences locally a structure close to an ensemble of thin and straight cylinders, or “sticks”. This is supported by the typical cytomorphology of fibrous astrocytes in WM, characterized by long, thin, and relatively unbranched structures in contrast to their protoplasmic counterparts in GM (Oberheim et al., 2009, Oberheim et al., 2012). The notion of a predominant intracellular contribution from neurites in both GM and WM in the human brain and other mammals has been expressed in previous reports, both from the DW-MRS literature ((Palombo et al., 2017), Lundell et al., 2020, Shemesh et al., 2017, Najac et al., 2014, Marchadour et al., 2012) as well as more recently from the DWI literature (Novikov et al., 2016). The high μFA values for intracellular water, tNAA, and tCr in GM support the notion that neurites dominate as a microstructural unit also in GM.

The high μFA in GM is in stark contrast to the overall low *macroscopic* anisotropy in GM obtained from DTI measurements, which reflects the overall lack of directionality in neurite propagation across the measurement volume, even at the millimeter scale. This has been alluded to in studies that investigated microscopic anisotropy in post-mortem tissue (Komlosh et al., 2007, Shemesh and Cohen, 2011, Nielsen et al., 2018, Ianuş et al., November 2017) as well as from DDE imaging studies in the human brain performed at low *b* values (Lawrenz and Finsterbusch, 2019), and is strong supported by this current study, with the added value of cross-validation between water and metabolite data. Noteworthy is that the μFA(water) values obtained in GM in a previous report (Lawrenz and Finsterbusch, 2019) are lower than those found in WM and lower than those reported here. At the *b* value in which these experiments were performed (total diffusion weighting of *b* < 1000 s/mm^2^) it is likely that signal from the more isotropic extracellular space in GM significantly contributed to the overall signal.

**Fig. 11:**
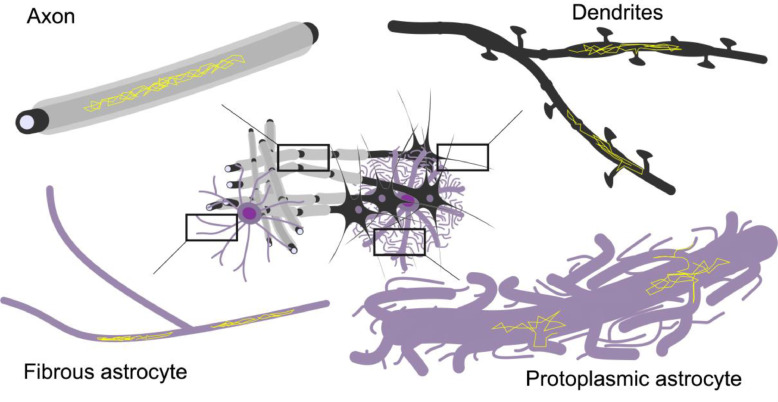
Schematic illustration of microstructural features such as deviation from propagation along a straight line and branching processes, that may affect microscopic diffusion metrics in neurons, fibrous, and protoplasmic astrocytes. Axons, dendrites and fibrous astrocytes are thin fibrous structures with few branches which may locally dictate highly anisotropic diffusion. Protoplasmic astrocytes are distinctly different, with highly branched and thicker processes that could entail a more isotropic diffusion, even at short diffusion times. In this qualitative illustration of different morphologies hypothezised to explain the differences in diffusion metrics across metabolites, different cells are not drawn to scale. Typical diameters of fibers are < 1 µm, while the processes of protoplasmic and fibrous astrocytes extend well beyond 100 µm.

The only exception to high intracellular μFA in GM in our measurements was the μFA(tCho). While extracellular tCho concentrations are very low and thus effects of transmembrane exchange can be excluded (Klein et al., 1993), this result can be interpreted in two possible ways. One is the lower tCho signal to noise ratio (SNR) for the single measurement, resulting in higher variability of the tCho signal across gradient orientations in the DDES results. The other possibility is that in GM, a significant fraction of the predominantly glial tCho is found in protoplasmic astrocytes. These astrocytes, found extensively in human GM, are highly branched cells, significantly more so than their fibrous counterparts in WM (Oberheim et al., 2012). It is plausible that the effective μFA(tCho) is low because the average diffusion length of tCho is comparable or higher than the distance between branching points on processes in protoplasmic astrocytes. Additional support for this explanation is also provided by the lower D_//_ of tCho in GM compared to the one in WM, mentioned in the following paragraph. The uniqueness of diffusion properties of tCho in human GM has been previously reported (Ingo et al., 2018), where the sub-diffusion index α (reflecting a distribution of diffusion coefficients that deviates from a Gaussian one towards a distribution that favors slower diffusion coefficients, or “sub-diffusion”) for tCho in GM was significantly lower than that of tCr and tNAA in GM and all three metabolites in WM. In Fig. 11 are shown schematic representations of microstructural features such as deviation from propagation along a straight line and branching processes, that may affect microscopic diffusion metrics at different diffusion lengths.

### Diffusion properties of the intracellular space are different in GM and WM

Correlations with tissue fraction and direct comparisons between data from OGM and PWM both indicate that the intracellular parallel diffusivity D_//_ obtained from metabolite DDES data was significantly different across tissue types as well as across metabolites within the same tissue type. D_//_ for all three metabolites was lower in OGM than in PWM, indicating an overall lower diffusivity in the intracellular space in GM. In good correspondence with this finding, D_//_ of water evaluated from the highest *b* value was lower in OGM than in PWM, and similarly to the metabolite data, D_//_ of water at the highest *b* value was strongly negatively correlated with GM fraction in the VOI. This tight correspondence between the tissue dependence of D_//_ of metabolites and D_//_ of water at the highest *b* value further corroborates our hypothesis that the extracellular water is effectively suppressed at *b* = 7199 s/mm^2^ and that the diffusional properties of water at this high *b* value reflect almost exclusively those of the intracellular water.

We used the strong correlation between D_//_ of both water and metabolites at the highest *b* value with tissue type fraction to estimate intracellular D_//_ of water and the three metabolites in pure GM and WM. Assuming that within the diffusion encoding times (~45 ms) in our experiments the diffusion length along the neurite represents a path along a straight-propagating unbranched fiber (< 15 μm), D_//_ can be seen as the cytoplasmic diffusion coefficient for the metabolites and the intracellular water. When branches and deviation from straight propagation occur within the diffusion length at a given *t_d_*, these geometric features will influence D_//_ as well (see Fig. 11). We observed a lower GM D_//_ for all metabolites, as well as for water at high *b*. Differences in D_//_ of tNAA between GM and WM may reflect differences in mitochondrial density between WM axonal fibers and GM neurons (Santuy et al., 2018) as well as morphological differences between long propagating WM axons and highly branched dendritic trees in cortical neurons. Differences in D_//_ of tCr, and mostly of tCho between the two regions may also reflect cytomorphological differences between astrocytes in both regions, as mentioned in the previous paragraph regarding µFA(tCho). Differences in metabolite D_//_ across tissue types are also consistent with tissue-specific metabolite ADC measurements obtained in previous DW-MRS studies (Ingo et al., 2018, Kan et al., 2012, Ercan et al., 2014, Najac et al., 2016, Ellegood et al., 2006, Ellegood et al., 2011).

Based on the D_//_ values of water and tNAA and their free diffusion coefficients at 37⁰C, we estimated the intracellular tortuosity experienced by water and tNAA, where the tortuosity of tNAA reflects exclusively that inside neurons. As vividly shown in Fig. 9E, the tortuosity values obtained for water and tNAA are remarkably similar within the same tissue type despite a 3-4 fold difference between the D_//_ for water and tNAA, and vary significantly between GM and WM. This means that the length scales probed by the different molecules differs over the time scale of the experiment. The intracellular tortuosity reflects all extrinsic factors that may slow down the mobility of molecules in the intracellular space, such as differences in viscosity, obstacles in the cytoplasm such as organelles, or undulations and varicosities (variations in diameter along the fiber). In light of this, the similarity between the tortuosity of water and tNAA in both tissue types suggests that despite the differences in their free and measured diffusivity, and thus the distances probed by the respective molecules, a similar coarse-grained environment is probed by both molecules over the diffusion encoding period. Similar relations have previously been shown for a number of metabolites and ions in macroscopic ADC measurements in rodents Ackerman and Neil (2010). This excellent agreement between two independent measurements suggests that the combined diffusion measurements of both water and metabolites can provide a valuable platform for validating models of water diffusion also on a microscopic scale in both GM and WM. We focused on the calculation of tortuosity of tNAA (T(tNAA)) because of the specificity to one particular cell type (neurons), as well as because of the relatively small contribution of the co-measured metabolite (NAAG) to the results. The tortuosity of tCr and tCho is more difficult to estimate without accurate information on the fractions of co-measured metabolites in tCr (Cr and PCr) and tCho (Cho, GPC and PCho), each with its specific molecular weight and concentration. Similarly, the values for free diffusivity of tCr and tCho require better assessment in phantom experiments with a realistic combination of co-measured metabolites that represents these combinations *in vivo* in GM and WM. We intend to pursue the investigation of the unique diffusion properties of tCr and tCho more thoroughly in future studies.

Values for D_//_(water) in WM we report here align well with earlier studies using different approaches for filtering extracellular and CSF contributions ((Veraart et al., 2019, Kunz et al., 2018, Budde et al., 2017, Dhital et al., 2019). Similar values have also been found in more model-driven analyses of conventional DWI data, which also suggest two-fold differences in axonal and dendritic intracellular diffusivities (Novikov et al., 2018). A recent study that used a similar model-driven approach suggested that intra-axonal diffusivity approached the value of free diffusion, with large variations across white matter (Howard et al., 2020). Estimating all contributions to the water signal from different tissue compartments provides a rather flat fitting landscape for selecting the right combination of fractions and diffusivities (Novikov et al., 2018, Lampinen et al., 2019, Jelescu et al., 2016), making the fitting procedure for compartment-specific microscopic diffusion metrics fairly unstable. The spatial resolution of conventional DWI is in general on the order of the cortical thickness, making direct measurements without contaminations from CSF and WM all but impossible. In our study, we operated on an even coarser resolution, many times over the cortical thickness. We demonstrated that taking into account tissue fractions within the VOI, it is possible to obtain consistent and reliable microstructural details on GM via correlation analyses. This can be easily extended to imaging studies where partial volume within DWI voxels can be obtained in a similar way to the one we used here (Koo et al., 2009).

D_//_ across metabolites within the same tissue type were also significantly different from one another. These differences were already observed in single diffusion encoding (SDE) DW-MRS experiments in humans and animal models (Palombo et al., 2017, Ingo et al., 2018, Kan et al., 2012, Najac et al., 2014, Najac et al., 2016, Ercan et al., 2015, Ligneul and Valette, 2017, Ronen et al., 2013). Interpretation of these values will have to take into account differences in cytoplasm across cell types, the potential effect of cytomorphological features, and molecular weight differences across the metabolites.

D_┴_(tNAA) in WM we report here is higher than the one we reported in a previous work, where we estimated the microscopic diffusion metrics of tNAA based on analysis of powder-averaged data (Lundell et al., 2020). There, reported values for D_┴_(tNAA) were between 0.011 and 0.024 µm^2^/ms. Underestimation and larger variance of µFA, equivalent to an underestimation of D_//_ and overestimation of D_┴_, may be attributed to a limited SNR (Lundell et al., 2020, Kerkelä et al., 2020). Our current measurements were conducted at longer TE, and with less averages compared to our previous study (Lundell et al., 2020), leading to a lower SNR in our present study. Simulation of noise propagation demonstrates an overestimation of D_┴_ in the case of a “stick” situation with negligible transverse displacements (Fig. 10). These estimates are slightly lower (50-75%) but in the same range of our D_┴_(tNAA) as estimated from our current data. A similar low D_┴_(water) was estimated in PWM but with a 2-3 fold increase in the more GM-rich OGM and sOGM voxels. This may reflect residual signal from the extracellular space, stemming either from non-complete filtering of the extracellular signal, or from repopulation of the extracellular pool via transmembrane exchange in the unmyelinated dendrites. Intracellular contributions from protoplasmic astrocytes, leading to the previously discussed lower μFA(tCho), may also play a role. The observation that D_┴_(tCho), and not D_┴_(tNAA), was significantly higher in OGM compared to PWM suggests that intracellular exchange across differently oriented and relatively short fiber segments through branching points, or non-negligible signal contribution from cell bodies are more prominent in glia than neurons. It is expected, however, that within the range of diffusion times and diffusion coefficients in our experiments, the effect of a nonfinite fiber radius on D_┴_ is small (Nilsson et al., 2017).

### Morphology of the extracellular space varies significantly between WM and GM

A significantly lower μFA in both PWM and OGM VOIs was observed at lower *b* values. Under the assumption that the contribution of the extracellular space to the measured signal decreases with increasing *b* value, this suggests that the extracellular space is less microscopically anisotropic than the intracellular space in both WM and GM, but that there is a significant degree of microscopic anisotropy in the extracellular space in WM, absent in GM. At the lowest *b*, water μFA is more than three times lower in OGM than in the PWM VOI, suggesting that the extracellular space in WM is highly anisotropic, or conversely, that the tortuosity in the direction perpendicular to the fiber direction significantly affects water diffusion in WM. This result is consistent with the fact that long axons are the dominant structural feature in WM and are relatively densely packed, resulting not only in a high intracellular anisotropy but also in a significant extracellular anisotropy. In previous studies it was suggested that in WM, D_//_ is higher in the intra-axonal space than in the extracellular space (Kunz et al., 2018, Dhital et al., 2019). Our results suggest otherwise, and it is plausible that CSF contamination in both VOIs, particularly in the OGM, adds a signal component with high isotropic diffusivity and biases our results.

### Data quality and limitations

With DDES now established in human settings, there are some experimental conditions which could be further optimized in future studies. Data here were averaged over three orthogonal planes and collected from relatively large VOIs. These conditions should approach the powder average condition, which assumes full orientational dispersion of fibers within the volume. Some residual order, however, could affect the observed signal modulations. Moreover, while intermediate angles carry information regarding oblate and prolate shapes (Nielsen et al., 2018), the maximum contrast for microscopic anisotropy is expected between the parallel and perpendicular conditions, as shown in Fig. 3, panels C and 3D. With this in mind, optimized sampling schemes for powder averaging with only the parallel and perpendicular conditions could be considered, such as the 5-design proposed by Jespersen (Jespersen et al., 2013), or the minimal protocol by Yang (Yang et al., 2018). The two protocols have recently been evaluated side-by-side with comparable performance (Kerkelä et al., 2020).

Our data fitting approach differs slightly from the original definition of µFA based on contrasting *b*^2^-terms (kurtosis) at low *b* values reflecting variance in mean displacements across either directions or domains (Jespersen et al., 2013, Lasič et al., 2014, Jespersen, 2014). While this provides a stringent theoretical description, larger *b* values might be needed to provide sufficient contrast-to-noise (see Fig. 3D), which in turn leads to the increasing influence of higher-order terms (Ianuş et al., November 2017). Here we followed an approach valid at all *b*, but under the assumption of mono-disperse gaussian domains, which was also applied in previous DDES studies (Shemesh et al., 2017, Vincent et al., 2020). Given the numerous potential interpretations of DDE/DDES data, a model-free representation of the data is given in the supplementary material (tables S2 and S3).

Finally, it is likely that the microstructural and diffusional properties in gray and white matter will vary across the brain and will be dictated by local myelo- and cytoarchitecture. This calls for expanding this type of investigations to other brain regions, or to incorporate DDES experiments in spatially resolved techniques such as magnetic resonance spectroscopic imaging (MRSI). An understanding of normative values in healthy tissue can provide important input for future evaluation of partial volumes of lesions in future DDES experiments.

### Possible time-dependence effects

While this study focused on the effects of microscopic anisotropy, the DDE experiment may entangle different temporal fingerprints of time-dependent diffusion phenomena, such as exchange across different domains (Lasič et al., 2011) or reflections from restrictive barriers (Mitra, 1995). These effects could bias measurements of microscopic anisotropy but could also provide rich additional information regarding microstructure or physiological processes. Our measurements were performed at relatively short mixing and gradient separation times (~5.3 ms and 45 ms), were kept constant throughout the experiments. In these conditions, our experiments do not capture the effect of typical exchange times in WM (in the order of seconds) reported in the literature (Lasič et al., 2011). Modulating the sequence timing towards shorter times may provide a contrast to faster exchange processes that may occur between branches or through the cell body of highly arborized cells such as protoplasmic astrocytes in GM or transmembrane exchange in non-myelinated fibers (Lampinen et al., 2016, Palombo et al., 2017, Williamson et al., 2019).

Time-dependent effects from restrictions could however potentially generate a difference between the signal intensity at parallel (θ=0°) and anti-parallel (θ=180°) directions with a lower signal in the latter (Mitra, 1995, Finsterbusch, 2011). We investigated the size of this contrast (see supplementary figure S3), but found no significant difference for the metabolites, in contrast to results reported in a recent DDES study in rodents. Higher SNR or shorter and stronger gradient pulses in the animal setting could reflect a different spatial scale and explain this difference. We did find a significant difference between S(θ=0°) and S(θ=180°) for water in the lowest *b* values in all VOIs. This suggests that water data over that range of *b* values are time-dependent. Time-dependence of microscopic diffusion metrics can be explained by several microstructural factors both in the extra- and intracellular environments, such as spatial disorder in axonal packing or variation in intracellular geometries (Fieremans et al., 2016). The lack of time-dependent effects on the diffusion metrics of metabolites and on those of water at high *b* values suggest that the time dependence captured by our experiment is mainly extra-cellular (Fieremans et al., 2016, Lee et al., 2018).

Measurements over a range of *b* values, mixing and diffusion times could indeed provide a more detailed view of the morphology of restrictions and the aforementioned exchange effects. Tuning the diffusion experiment to the expected phenomena with e.g. optimized gradient waveforms could be a promising approach for detecting independent effects that affect microscopic anisotropy and other local diffusion metrics (Lundell et al., 2019, Lundell and Lasič, 2020). Such data could also challenge more intricate biophysical models featuring multiple geometrical features, such as spherical cell bodies, organelles or connected and arborizing fibrous structures of particular relevance for the study of neuronal structures in GM and for the characterization of different types of astrocytes across the brain (Williamson et al., 2019, Ronen et al., 2013, Palombo et al., 2016, Palombo et al., 2020).

## Conclusion

We presented here a comprehensive approach for combining water and metabolite DDES to investigate the local microstructural and cytoplasmic features of extra- and intracellular spaces in the human brain. We demonstrated the usefulness of this approach by shedding light on differences as well as similarities in local anisotropy and cytoplasmic properties in different cell types in gray and white matter, and inferred on differences in local geometry between the two tissue types. This approach can be extended to combine other microstructural probes, such as *b* tensor encodings, and be further expanded to investigate time dependent effects on intra- and extracellular diffusion properties to further characterize brain tissue microstructure.

## Declaration of Competing Interest

None.

## Appendix Supplementary materials

Image, application 1
