# Supplementary material for: Compartmental diffusion and microstructural properties of human brain gray and white matter studied with double diffusion encoding magnetic resonance spectroscopy of metabolites and water
Source: Neuroimage. 2021 Jul 1;234:117981. doi: 10.1016/j.neuroimage.2021.117981 (PMC8204266; doi:10.1016/j.neuroimage.2021.117981)
Supplement: Supplementary file 1 [file mmc1.docx]

**Supplementary information**

**Compartmental diffusion and microstructural properties of human brain gray and white matter studied with double diffusion encoding magnetic resonance spectroscopy of metabolites and water**

Henrik Lundell*^#1^, Chloé Najac*^2^, Marjolein Bulk^2^, Hermien E. Kan^2^, Andrew G. Webb^2^, Itamar Ronen^2^

^1^Danish Research Centre for Magnetic Resonance, Copenhagen University Hospital Hvidovre, Centre for Functional and Diagnostic Imaging and Research, Kettegaards Allé 30, 2650 Hvidovre, Denmark

^2^C.J. Gorter Center for High Field MRI, Department of Radiology, Leiden University Medical Center, Albinusdreef 2, 2333 ZA Leiden, The Netherlands

**Multi-compartmental effects**

A simple model can serve as a hypothetical model of the contributions of intracellular, extracellular, and CSF components of the signal at different *b* values. Simulated signals are shown in figure S1. Here, the intracellular diffusivity is modeled as a “stick” with zero axial diffusivity, the extracellular space as an anisotropic tensor, and CSF as an isotropic tensor. Examples of representative diffusivities are taken from figure 4 in Novikov et al^1^ and for WM were set to : intracellular D_//_/D_┴_ = 2/0 µm^2^/ms, extracellular D_//_/D_┴_ = 2/0.5 µm^2^/ms; and for GM set to: intracellular D_//_/D_┴_ = 1/0 µm^2^/ms, extracellular D_//_/D_┴_ = 0.75/0.5 µm^2^/ms. CSF diffusivity was set to 3 µm^2^/ms.


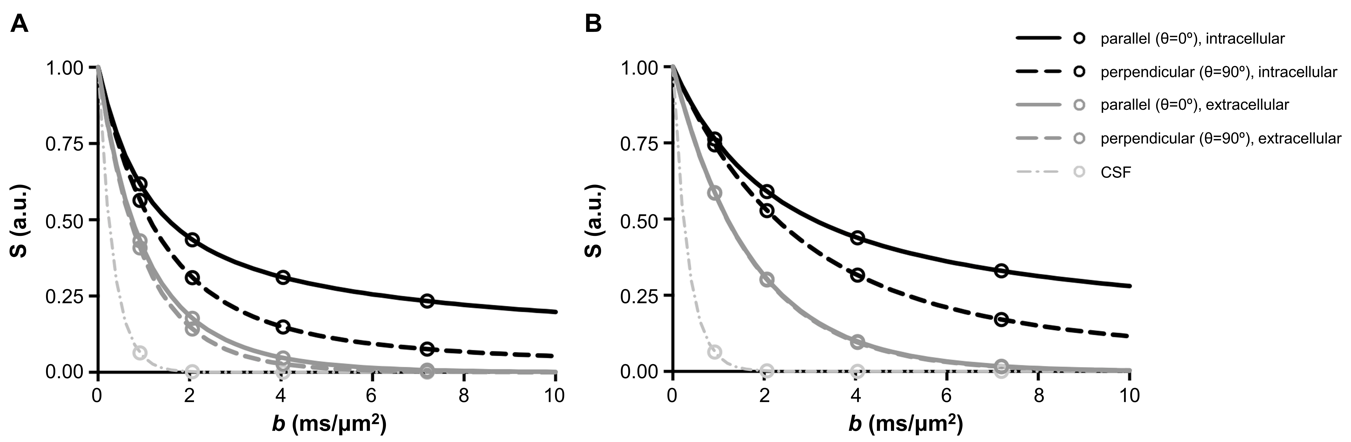
***Figure S1****: Simulated powder averaged signals for the parallel (solid lines) and perpendicular (dashed lines) conditions for three different signal components in WM (A) and GM (B). All signals are normalized. The b values used in the water acquisitions are shown with markers. At the highest b value, the intracellular component dominates and in all but the lowest b value, the CSF contribution is suppressed below <0.25%.*

**Effect and correction of contaminating gradient fields - phantom data and simulations**

Cross-terms between the diffusion gradients and static background gradients or imaging gradients (such as the crushers and slice-selection gradients) may bias the measurement of D_//,_ D_┴_ and μFA. To investigate this effect we acquired all data with both positive and negative diffusion gradient polarities. The geometric mean of the signal over both conditions was then calculated which cancels out cross-terms to first order in *b* value. Our sequence and approach were validated *in vitro* using the “BRAINO” phantom (GE Medical Systems, Milwaukee, WI, USA). Water DW-spectra were acquired with two *b* values (0 and 1111 s/mm^2^) and both diffusion gradient polarities. For each condition, the water signal was calculated as the peak integral. As illustrated in figure S2A, the behavior of the water signal as a function of θ changes with gradient polarities and as expected becomes independent of θ when taking the geometric mean of the signal acquired with both polarities. This demonstrates that the effects of cross-terms between diffusion and imaging gradients are then corrected. We further validated this using Matlab simulations calculating the signals from *b* tensors with and without contributions from background or imaging gradients. Diffusion and imaging gradients were extracted from a simulation of the sequence on the scanner software as described earlier. This showed that all cross-terms between diffusion and imaging gradients are canceled as well as cross-terms with spatially constant background gradients (figure S2B and C). However, the effects spatially varying microscopic background gradients on the length scale of the diffusion pathway are not cancelled.

***
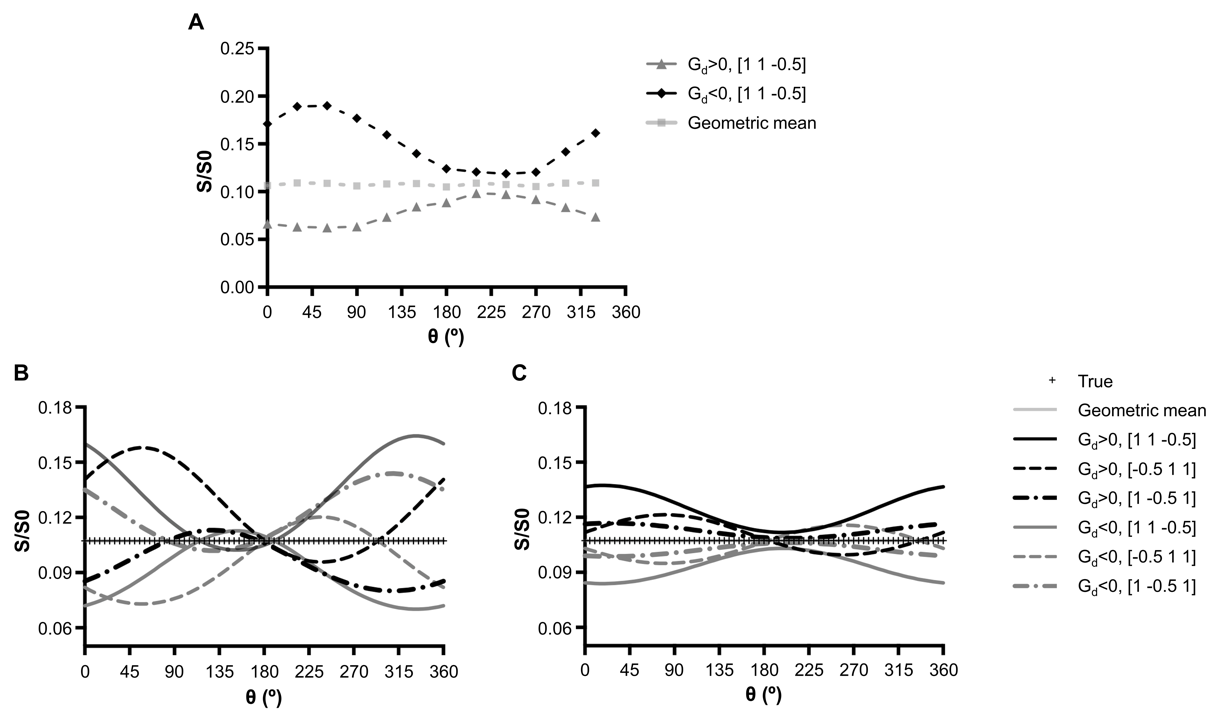
Figure S2****: (A) Illustration of the effect of cross-terms between the diffusion gradients and imaging gradients in BRAINO (GE Medical Systems, Milwaukee, WI, USA) phantom is illustrated. This was further investigated using simulations showing the effect of cross-terms between (A) diffusion gradients and imaging gradients or (C) diffusion gradients and background gradients (G_background_=[1 1 0] and 10mT/m).*

**Time dependence**

If the mixing time in a DDE experiment is short compared to the characteristic length of a restriction a difference in signal between the anti-parallel and parallel conditions will be observed^2,3^. Diffusion in the proximity of barriers is likely to change direction from boundary reflections, leading to a larger signal attenuation when the encoding direction change is in the anti-parallel condition. In contrast, the anti-parallel condition is velocity compensated which means that ballistic intravoxel incoherent motion (IVIM) from e.g. disperse blood flow in capillaries, will be rephased leading to the opposite pattern^4,5^. Pairwise comparison of the two conditions is shown in figure S3. We observe significantly lower anti-parallel signals in the order of ~1% signal difference in the lower b values of the water acquisition in both PWM and OCC which support apparent effects from restrictions/reflections rather than IVIM effects.

***
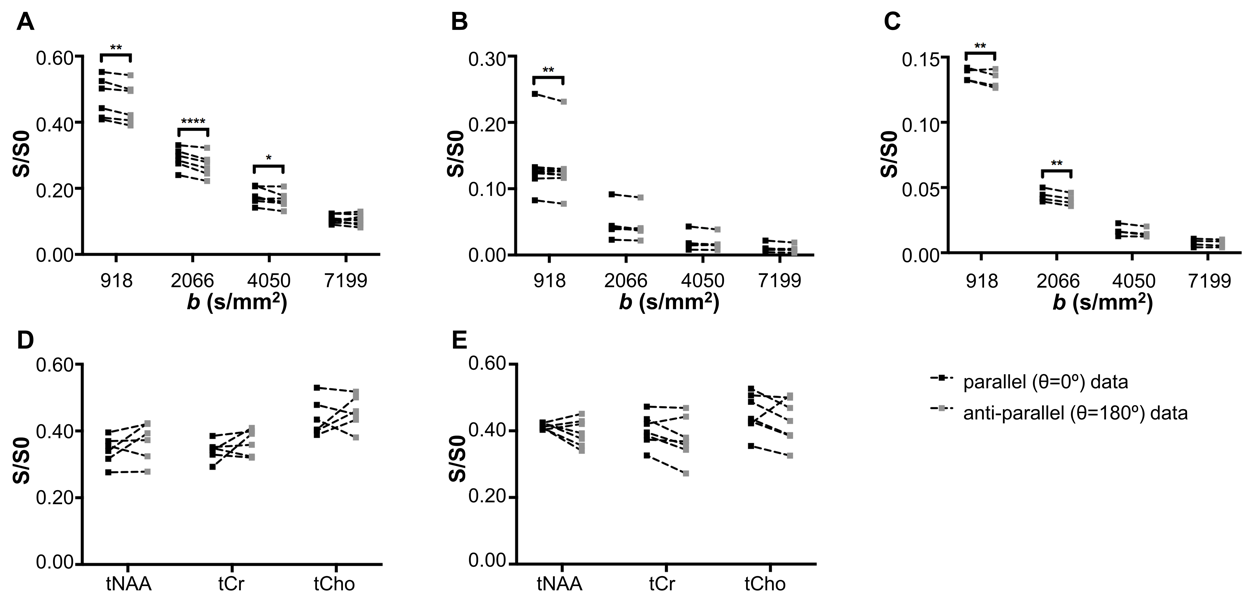
Figure S3****: Comparison of the difference in signal quantified with parallel (θ=0º) and anti-parallel (θ=180º) directions for water in (A) PWM, (B) OGM and (C) OGMs VOIs as well as for metabolites data in (D) PWM and (E) OGM VOIs at different b values. Connected lines (dashed lines) between data points (square) connect data from each participant’s data. Significant differences between parallel and anti-parallel (represented with *) lower b values were observed in both VOIs for water data. * p<0.05, ** p<0.005 and *** p<0.001.*

**Small OGM VOI data**

Additional water data from 4 participants with a smaller VOI was collected in OGM to achieve a larger relative fraction of gray matter. The data is included in figure 8 in the main paper. Tissue volume fractions and fitted model parameters are shown in table S1.

| **Gray Matter (GM, %)**  **White Matter (WM, %)**  **Cerebrospinal Fluid (CSF, %)** | 67.7±4.5  19.8±3.9  12.5±6.4 | | | |
| --- | --- | --- | --- | --- |
|  |  |  |  |  |
|  | ***b*=918 s/mm^2^** | ***b*=2066 s/mm^2^** | ***b*=4050 s/mm^2^** | ***b*=7199 s/mm^2^** |
| **Fitted S_0_** | 1.00 | 0.38±0.03 | 0.15±0.01 | 0.07±0.02 |
| **D_//_ (μm^2^/ms)** | 2.91±0.15 | 1.85±0.15 | 1.37±0.13 | 1.18±0.13 |
| **D_┴_ (μm^2^/ms)** | 1.84±0.10 | 0.79±0.09 | 0.35±0.09 | 0.18±0.08 |
| **μFA** | 0.28±0.06 | 0.49±0.06 | 0.69±0.10 | 0.82±0.10 |

***Table S1****: Volume fraction (%, mean±s.d.) of WM, GM, and CSF and fitted model parameters for the water data (mean±s.d.) in the sOGM VOI.*

**Simple representation of signal**

A simple representation of the signal can serve as an input for alternative interpretations. The relevant information (disregarding possible effects from time dependence) is the offset and amplitude of the angular modulation. In the tables S2 and S3 we state the values from fitting the following equation to the normalized data as suggested in earlier studies^6,7^:

$$S(\theta) = A+B\cdot\cos2\theta$$

|  |  | **tNAA** | **tCr** | **tCho** |
| --- | --- | --- | --- | --- |
| **A** | **PWM** | 0.310±0.047 | 0.297±0.045 | 0.406±0.068 |
|  | **OGM** | 0.380±0.019 | 0.365±0.053 | 0.443±0.063 |
| **B** | **PWM** | 0.038±0.011 | 0.042±0.011 | 0.024±0.009 |
|  | **OGM** | 0.026±0.008 | 0.025±0.012 | 0.004±0.016 |

***Table S2****: Phenomenological representation of the signal offset A and modulation amplitude B for metabolites at b* = 7199 s/mm^2^*.*

|  |  | ***b*=918 s/mm^2^** | ***b*=2066 s/mm^2^** | ***b*=4050 s/mm^2^** | ***b*=7199 s/mm^2^** |
| --- | --- | --- | --- | --- | --- |
| **A** | **PWM** | 0.452±0.058 | 0.243±0.024 | 0.125±0.014 | 0.064±0.008 |
|  | **OGM** | 0.135±0.045 | 0.041±0.020 | 0.015±0.008 | 0.006±0.003 |
|  | **sOGM** | *0.435±0.055* | *0.230±0.026* | *0.120±0.018* | *0.061±0.007* |
| **B** | **PWM** | 0.022±0.005 | 0.039±0.006 | 0.043±0.006 | 0.033±0.005 |
|  | **OGM** | 0.002±0.001 | 0.003±0.002 | 0.003±0.002 | 0.003±0.001 |
|  | **sOGM** | *0.019±0.003* | *0.035±0.006* | *0.040±0.007* | *0.030±0.002* |

***Table S3****: Phenomenological representation of the signal offset A and modulation amplitude B for water at different b values.*

1. Novikov DS, Veraart J, Jelescu IO, Fieremans E. Rotationally-invariant mapping of scalar and orientational metrics of neuronal microstructure with diffusion MRI. *Neuroimage*. 2018;174:518-538. doi:10.1016/j.neuroimage.2018.03.006

2. Mitra PP. Multiple wave-vector extensions of the NMR pulsed-field-gradient spin-echo diffusion measurement. *Phys Rev B*. 1995;51(21):15074-15078. doi:10.1103/PhysRevB.51.15074

3. Finsterbusch J. The parallel-antiparallel signal difference in double-wave-vector diffusion-weighted MR at short mixing times: A phase evolution perspective. *J Magn Reson*. 2011;208(1):114-121. doi:10.1016/j.jmr.2010.10.012

4. Le Bihan D, Breton E, Lallemand D, Grenier P, Cabanis E, Laval-Jeantet M. MR imaging of intravoxel incoherent motions: application to diffusion and perfusion in neurologic disorders. *Radiology*. 1986;161:401-407. doi:10.1148/radiology.161.2.3763909

5. Ahlgren A, Knutsson L, Wirestam R, et al. Quantification of microcirculatory parameters by joint analysis of flow-compensated and non-flow-compensated intravoxel incoherent motion (IVIM) data. *NMR Biomed*. 2016;29(5):640-649. doi:10.1002/nbm.3505

6. Vincent M, Palombo M, Valette J. Revisiting double diffusion encoding MRS in the mouse brain at 11.7T: which microstructural features are we sensitive to? *arXiv*. 2019:1908.00317.

7. Shemesh N, Rosenberg JT, Dumez JN, Grant SC, Frydman L. Distinguishing neuronal from astrocytic subcellular microstructures using in vivo Double Diffusion Encoded 1H MRS at 21.1 T. *PLoS One*. 2017;12(10):1-19. doi:10.1371/journal.pone.0185232
